# Effects of high-quality nursing care on psychological outcomes and quality of life in patients with hepatocellular carcinoma: A protocol of systematic review and meta-analysis

**DOI:** 10.1097/MD.0000000000021855

**Published:** 2020-08-28

**Authors:** Lei Zhang, Xuan Zhang, Zhaokun Cui, Lijuan Zhou, Kai Qu, Nannan Wang

**Affiliations:** a Department of Oncology, The People's Hospital of Taizhou, Taizhou, Jiangsu Province; b Digestive Endoscopy Center, Liaocheng People's Hospital; c Department of Anesthesiology, Liaocheng Infectious Diseases Hospital, Liaocheng, Shandong Province; d Department of Nursing, The People's Hospital of Taizhou, Taizhou, Jiangsu Province; e Department of Hepatobiliary Surgery, The First Affiliated Hospital of Xi’an Jiaotong University, Xi’an, Shaanxi Province; f The Sixth Ward of Hepatology Department, Qingdao Sixth People's Hospital, Qingdao, Shandong Province, PR China.

**Keywords:** hepatocellular carcinoma, high quality nursing care, meta–analysis, psychological disorder, quality of life

## Abstract

**Background:**

High quality nursing care (HQNC) has been reported to effectively prevent psychological disorders and improve the quality of life (QoL) in patients with hepatocellular carcinoma (HCC) during the treatment. However, the exact effect of HQNC remains controversial. This systematic review will be aimed to assess the effectiveness of HQNC on psychological disorders and QoL in patients with HCC.

**Methods:**

Eligible prospective controlled clinical trials were searched from Google Scholar, Medline, Excerpt Medica Database (Embase), PubMed, Web of Science (WOS), Cochrane Library, China Scientific Journal Database (CSJD), China National Knowledge Infrastructure (CNKI), Chinese BioMedical Database (CBM) and Wanfang Database. Papers in English or Chinese published from January 2000 to July 2020 will be included without any restrictions. The clinical outcomes including psychological outcomes, QoL, and adverse events of HQNC in patients with HCC were systematically evaluated.

Study selection and data extraction will be performed independently by two reviewers. Stata 14.0 and Review Manager 5.3 were used for data analysis. Methodological quality for each eligible study will be assessed by using Cochrane risk of bias tool. Subgroup and meta-regression analysis will be carried out depending on the availability of sufficient data.

**Results:**

The results of this systematic review will be published in a peer-reviewed journal.

**Conclusion:**

The results of this study may provide helpful evidence of HQNC on psychological effects and QoL in patients with HCC.

**INPLASY registration number:**

INPLASY202070096.

## 1 Introduction

Hepatocellular carcinoma (HCC) is the seventh most commonly diagnosed malignancy and the second most frequent cause of cancer-related death.^[1,2]^ According to global cancer statistics, about 841,080 newly diagnosed cases (4.7% of all sites) and 781,631 deaths (8.2% of all sites) occurred worldwide in 2018.^[1,2]^ The main factors that cause HCC are hepatitis B virus and hepatitis C virus infection, excessive alcohol consumption, contact or consumption of Aspergillus toxins as well as various metabolic disorders.^[3–5]^ Despite the improvement of diagnostic and therapeutic methods in the past decades, the prognosis of HCC remains unsatisfactory.^[6–8]^ More than half HCC patients already have advanced or metastatic lesions when diagnosed, due to the lack of noticeable clinical symptoms at early stage, and the 5-year survival rate of advanced HCC patients was less than 17%.^[9,10]^ Currently, the clinical treatment of HCC mainly includes chemotherapy, radiotherapy, and surgery.^[9]^ However, most patients who received these traditional treatments also experience more psychological disorders, such as depression and anxiety.^[11–15]^ In addition, the unpleasant side effects of HCC treatment are also seriously affect the quality of life (QoL) of patients.^[15–17]^

**Figure 1:**
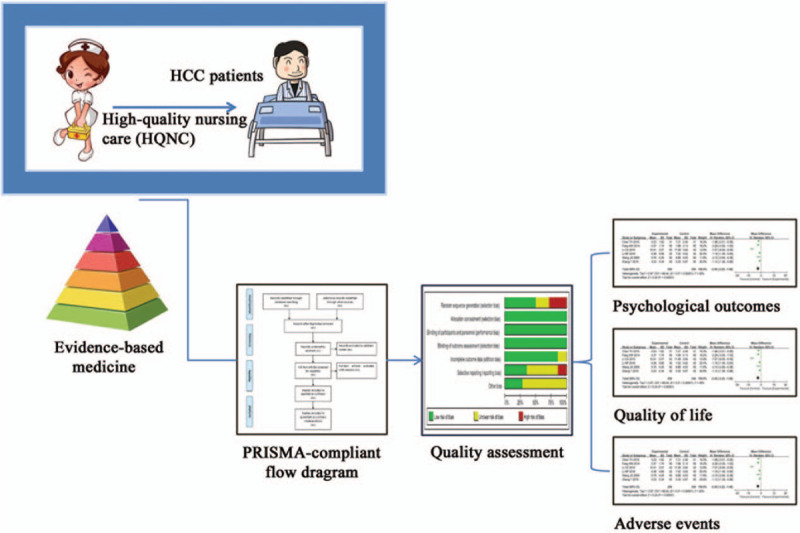
Work flow of the present study.

High quality nursing care (HQNC) has been reported to effectively prevent psychological disorders and improve the QoL in patients with HCC in several studies.^[18–23]^ Unfortunately, no study has systematically assessed these effects of HQNC for HCC. Therefore, in this study, we will systematically evaluate the preventive effect of HQNC on psychological disorders, and the improvement effect of HQNC on QoL in patient with HCC through the meta-analysis, in order to provide scientific reference for the design of future clinical trials (Work flow of the present study, Fig. 1).

## 2 Study aim/objective

The aim of our study is to propose a protocol for a systematic review and meta-analysis to assess the effectiveness of HQNC on psychological outcomes and QoL in patients with HCC.

## 3 Methods

The protocol of systematic review and meta-analysis will be reported according to the Preferred Reporting Items for Systematic Reviews and Meta-Analyses Protocols (PRISMA-P) guidelines.^[24]^ Our protocol has been registered on the International Platform of Registered Systematic Review and Meta-Analysis Protocols (INPLASY). The registration number was INPLASY202070096 (DOI number is 10.37766/inplasy2020.7.0096, https://inplasy.com/inplasy-2020-7-0096/). No ethic approval is required for this study, because all the data will be extracted from previous published studies.

### 3.1 Eligibility criteria

#### 3.1.1 Study type

All available randomized controlled trials and high-quality prospective cohort studies that assessed the effectiveness of HQNC on psychological outcomes and QoL in patients with HCC will be included in this systematic review.

#### 3.1.2 Participants or population

All patients with HCC are clinically diagnosed depression disorder or poor QoL will be included in this study, without restrictions of country, race, gender, etc.

#### 3.1.3 Intervention

In the experimental group, all patients must receive HQNC for the psychological disorders or for improving the QoL.

#### 3.1.4 Comparator

The control intervention can be any therapies, except HQNC.

#### 3.1.5 Exclusion criteria

Articles without sufficient available data, noncomparative studies, nonpeer reviewed articles, literature reviews, meta-analysis, commentaries, case reports and series, meeting abstracts, letter to the editor, editorials, and other unrelated studies will be all excluded from analysis.

### 3.2 Information sources

Electronic databases including Google Scholar, Medline, Excerpt Medica Database (Embase), PubMed, Web of Science (WOS), Cochrane Library, China Scientific Journal Database (CSJD), China National Knowledge Infrastructure (CNKI), Chinese BioMedical Database (CBM), and Wanfang Database, will be systematically searched for eligible studies from January 2000 to July 2020. Language is limited with English and Chinese.

### 3.3 Search strategy

To perform a comprehensive and focused search, experienced systematic review researchers will be invited to develop a search strategy. The plan searched terms are as follows: “liver cancer” or “hepatocellular cancer” or “LC” or “HC” or “HCC” and “depression” or “anxiety” or “psychological disorder” or “quality of life” or “adverse events” and “nursing care” or “advanced nursing care” or “high quality nursing care” or “psychological care” et al. The detailed sample of search strategy for PubMed database is shown in Table 1. Similar search strategies will be modified and used for the other databases.

**Table 1:**
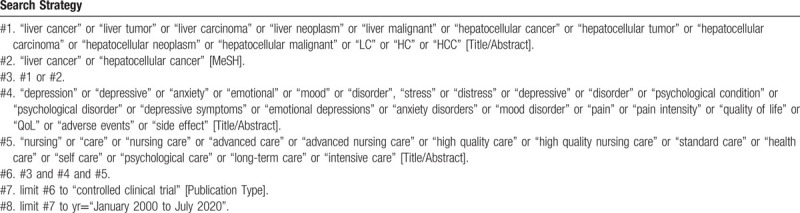
Searching strategy in PubMed.

### 3.4 Types of outcomes

#### 3.4.1 Main outcomes

The primary outcomes will include:

- Depression, which is measured by the Hamilton Depression Rating Scale or any relevant scales.
- Anxiety, which is measured by the Hamilton Anxiety Rating Scale or other tools.
- QoL which is assessed by 36-Item Short Form Health Survey or any other associated scales or scores.

#### 3.4.2 Additional outcomes

The secondary outcomes comprise of pain intensity and adverse events.

- Pain intensity can be assessed by visual analog scale or other scales.
- Any expected or unexpected adverse events, which are measured according to World Health Organization (WHO) standards, will be also measured.

### 3.5 Data collection and analysis

We will adopt the measures described in the Cochrane Handbook for Systematic Reviews of Interventions to pool the evidence.^[25]^

#### 3.5.1 Study selection

**Figure 2:**
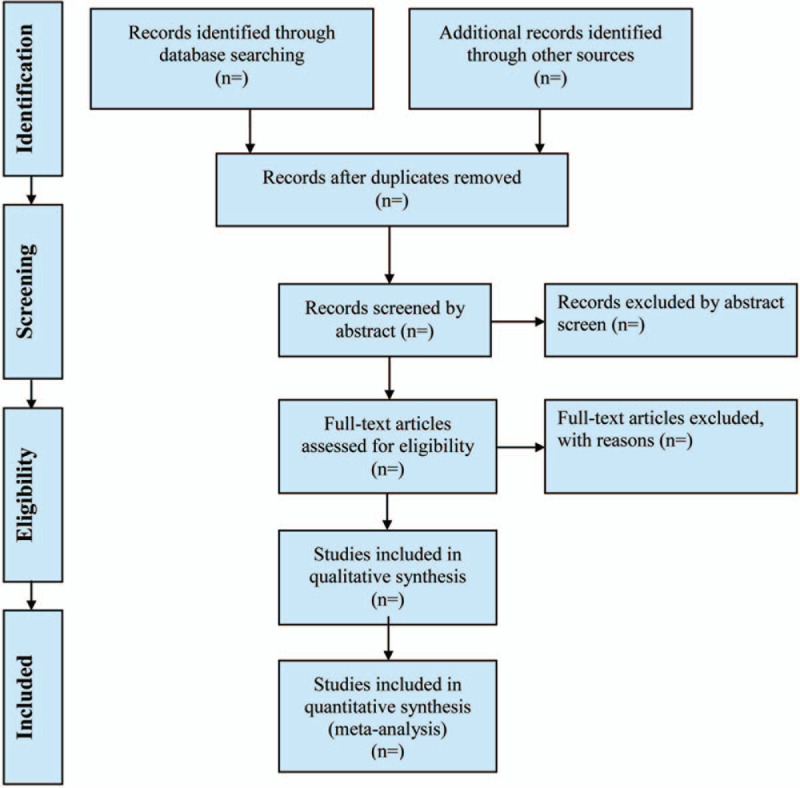
Study selection process for the meta-analysis.

Two experienced authors (Zhang L and Zhang X) will be reviewed independently to identify potential trials by assessing the titles and abstracts. The full text will be further reviewed to exclude irrelevant studies or determine potential eligible studies. Disagreements between the 2 authors will be resolved by discussing with the third investigator (Cui ZK). Endnote X7 software will be used for literature managing and records searching. A Preferred Reporting Items for Systematic Reviews and Meta-Analyses-compliant flow chart (Fig. 2) will be used to describe the selection process of eligible literatures. Excluded studies and the reasons for exclusion will be listed in a table.

#### 3.5.2 Data extraction

Two investigators (Zhang L and Zhang X) will be responsible for the data extraction independently. The following data will be extracted from eligible literatures:

- Study characteristics: first author's name, year of publication, country of study, sample size, study methods (such as randomization, blinding, etc), periods of data collection and follow-up duration, et al.
- Participant characteristics: tumor stage (staging of the tumor according to the AJCC TNM classification for esophageal cancer), age, gender, ethnicity, pathology diagnosis, pathologic tumor size, inclusion and exclusion criteria, et al.
- Interventions: intervention methods and duration of intervention, et al.

#### 3.5.3 Dealing with missing data

When any data are missing or insufficient, we will contact primary study authors by using email. If those relevant data are not acquired, we will only analyze the available data, and discuss its impact as a limitation.

### 3.6 Quality assessment

Two experienced authors (Zhang L and Zhang X) will assess the risk of bias for each eligible trial according to the guidance of the Cochrane Handbook for Systematic Review of Interventions independently.^[25,26]^ This tool comprises of 7 items including selection, selection, performance, detection, attrition, reporting and other bias, and each item is further divided as 3 different levels: high, unclear, or low risk of bias. EPHCC guidelines will be used to assess the risks of non- randomized controlled trials.^[27]^ Any disagreements will be resolved via discussion with a third researcher (Zhou LJ).

### 3.7 Data synthesis and analysis

Stata 14.0 (Stata Corp., College Station, TX) and Review Manager 5.3 (Nordic Cochran Centre, Copenhagen, Denmark) statistical software were used for statistical analyses. Continuous data will be presented as standardized mean difference with their 95% confidence intervals, and dichotomous data will be recorded as risk ratio with 95% their confidence intervals. A 2-tailed *P* < .05 was considered statistically significant. Cochrane *Q* test and *I*^2^ statistics were used to assess heterogeneity among the included clinical trials.^[28]^ If *P > *.1 or *I*^*2*^ < 50%, a fixed effects model was used for the meta-analysis; otherwise, a random effects model was used.

### 3.8 Subgroup and meta-regression analysis

If the data are available and sufficient, subgroup and meta-regression analysis will be conducted to explore the source of heterogeneity with respect to age, gender, tumor stage, intervention types, study quality, location, and treatment duration.

### 3.9 Sensitivity analysis

Sensitivity analysis will be carried out to assess the reliability and robustness of the aggregation results via eliminating trials with low quality or high bias risk. A summary table will report the results of the sensitivity analyses.

### 3.10 Publication bias analysis

We will detect publication biases and poor methodological quality of small studies using funnel plots if 10 or more studies are included in the meta-analysis. Begg and Egger regression test will be utilized to detect the funnel plot asymmetry.^[29–31]^ If reporting bias is suspected, we will consult the study author to get more information. If publication bias existed, a trim-and-fill method should be applied to coordinate the estimates from unpublished studies, and the adjusted results were compared with the original pooled RR.^[32]^

### 3.11 Evidence evaluation

The evidence grade will be determined by using the guidelines of the Grading of Recommendations, Assessment, Development, and Evaluation (GRADE). The quality of all evidence will be evaluated as high, moderate, low, and very low levels, respectively.^[25,33]^

### 3.12 Dissemination plans

We will disseminate the results of this systematic review by publishing the manuscript in a peer-reviewed journal.

## 4 Discussion

HCC is a highly malignant tumor, and current treatment methods only have a modest survival benefit.^[9]^ In addition, most patients with HCC also suffer from depression disorder.^[11]^ Although several managements can help relieve psychological disorder and improve QoL in patients with HCC,^[18,20,21,23]^ it is not always effective for some patients. Therefore, therapies that could significantly improve psychological health condition and living quality are what we need to pursue now.

### 4.1 Strengths and limitations of this study

Even though there was statistical analysis of published clinical trials,^[18–23]^ the exact therapeutic effects of HQNC on psychological disorders and QoL in patients with HCC were remains controversial. This systematic review will provide a helpful evidence for clinicians to formulate the best treatment strategy for HCC patients with psychological disorder and poor QoL, and also provide scientific clues for researchers in this field.

The systematic review will also have some limitations. There may be a language bias with the limitation of English and Chinese studies. In addition, Clinical heterogeneity may exist for different tumor stage and ages of HCC patients, and duration of HQNC.

## Author contributions

**Conceptualization:** Lijuan Zhou, Nannan Wang, Lei Zhang.

**Funding acquisition:** Kai Qu.

**Investigation:** Lei Zhang, Xuan Zhang, Zhaokun Cui, Lijuan Zhou.

**Methodology:** Lei Zhang, Xuan Zhang, Zhaokun Cui, Lijuan Zhou.

**Project administration:** Lei Zhang, Nannan Wang.

**Supervision:** Lei Zhang, Nannan Wang.

**Writing – original draft:** Lei Zhang, Xuan Zhang, Zhaokun Cui, Lijuan Zhou.

**Writing – review & editing:** Lei Zhang, Kai Qu, Nannan Wang.
